# Primary care virtual resource use prior and post COVID-19 pandemic onset

**DOI:** 10.1186/s12913-022-08790-w

**Published:** 2022-11-18

**Authors:** Jolie N. Haun, Vanessa Panaite, Bridget A. Cotner, Christine Melillo, Hari H. Venkatachalam, Christopher A. Fowler, William Lapcevic, Amy C. Alman, Dustin D. French, Brian Zilka, William Messina

**Affiliations:** 1 Research and Development Service, James A. Haley Veterans’ Hospital, 8900 Grand Oak Circle, Tampa, FL 33637 USA; 2 College of Public Health, University of South Florida, Tampa, FL 33620 USA; 3 Department of Psychology, University of South Florida, Tampa, FL 33620 USA; 4 Department of Anthropology, University of South Florida, Tampa, FL 33620 USA; 5 Department of Psychiatry and Behavioral Neurosciences, University of South Florida, Tampa, FL USA; 6 Center for Health Services and Outcomes Research and Department of Ophthalmology and Medical Social Sciences, Feinberg School of Medicine, Northwestern University, Chicago, IL USA; 7 U.S. Department of Veterans Affairs, Health Services Research and Development, Chicago, IL USA; 8 James A. Haley Veterans’ Hospital and Clinics, Tampa, FL 33637 USA

**Keywords:** Veteran, Virtual care, Primary care, COVID-19, Data extraction

## Abstract

**Background:**

The COVID-19 pandemic has been a catalyst for rapid uptake of virtual care through the use of virtual health resources (VHR). In the Department of Veterans Affairs (VA) Healthcare System, virtual care has been critical to maintaining healthcare access for patients during COVID-19. In the current study we describe primary care patient aligned care team (PACT) VHR use patterns within one VA medical center (i.e., hospital facility and five community-based outpatient clinics) pre- and post-COVID-19 onset.

**Methods:**

VHR provider and patient use data from 106 individual PACTs were extracted monthly between September 2019 to September 2020. Data were extracted from VHA web-based project application and tracking databases. Using longitudinal data, mixed effect models were used to compare pre- and post-COVID onset slopes.

**Results:**

Findings highlight an increase in patient users of secure messaging (SM) and telehealth. The rate of utilization among these patients increased for SM but not for telehealth visits or online prescription refill (RxRefill) use. Finally, VetLink Kiosk check ins that are done at in person visits, diminished abruptly after COVID-19 onset.

**Conclusions:**

These data provide a baseline of VHR use at the PACT level after the initial impact of the COVID-19 pandemic and can inform healthcare delivery changes within the VA systems over time. Moreover, this project produced a data extraction blueprint, that is the first of its kind to track VA VHR use leveraging secondary data sources.

**Supplementary Information:**

The online version contains supplementary material available at 10.1186/s12913-022-08790-w.

## Callout box

What is already known on this topic?

- VA has integrated a variety of VHR into daily practice.
- Utilization of virtual health care resource uptake in VHA has varied over time, and efforts are needed to optimize uptake.

What this study adds?

- The first VA study to evaluate trajectories of utilization of VHR changes pre-post COVID 19 onset.
- VHA uptake increased, however rate of utilization among users largely has not changed, and efforts to optimize proactive integrated use are still needed.
- Data extraction blueprint is shared for use to support monitoring virtual healthcare resource use over time.

## Introduction

The COVID-19 pandemic has been a catalyst for rapid uptake of virtual care, requiring virtual healthcare resources (VHR) (e.g., secure messaging, mobile health applications, telehealth) to provide safe, timely, and remotely accessible healthcare [1, 2]. In the Department of Veterans Affairs (VA) Healthcare System, virtual care has been critical to maintaining healthcare access for patients during COVID-19 [3]. The VA is a leader in development of integrated VHR [4], however, the majority of outpatient primary and specialty care continued to be in-person through February 2020 [3]. Furthermore, the emergence of the VA’s priorities in providing *connected care,* has pushed proactive integrated VHR use into the forefront. Proactive integrated VHR use is defined as a self-initiated approach to coordinated use of applicable VHR.

systems for the purposes of coordinating and delivering timely high-quality patient-centered care. We contend the proactive integrated use of VHR will maximize the capacity for using VHR to efficiently deliver remote, yet accessible healthcare.

In the VA, the patient-aligned care team (PACT) initiative was implemented between 2010 and 2014 to achieve team-based primary care, improve access, and provide comprehensive care management for more than 8 million veterans’ primary care needs [5]. The PACT model delivers primary care through a team of professionals, typically comprised of a physician, nurse, and other clinical and clerical associates, referred to as a PACT teamlet. PACT providers most often used the telephone to communicate with their patients compared to other VHR (i.e., secure messaging, mobile health applications, and telehealth). The various VHR made available by the VA allow patients to meet with their PACT, upload their health data for review, co-develop treatment plans, and refill prescriptions, without having to be present in the same location as their PACT. PACT health care services have extended beyond in-person delivery since its inception [6]. However, adoption and use of VHR such as telehealth has been slow, with far fewer patient visits conducted through telehealth than in person in early 2020 [3, 7, 8].

The VA Office of the Inspector General conducted a review of facility leaders’ assessments of healthcare operations affected by COVID-19 from March 11 through June 15, 2020. Facility leaders reported increases in use of VHR from pre-pandemic levels, such as telephone, VA Video Connect, or FaceTime, to provide care [9]. Leveraging existing infrastructure and prior planning, rapid increases in telehealth use occurred after the onset of COVID-19 [6]. While increases in VHR use within the VA have occurred due to public health restrictions implemented during the pandemic [3, 10], it is not clear exactly how use of VHR by PACTs has changed. We expect that recent changes would positively impact VHR use patterns; however, it is not clear that increased use has resulted in proactive integrated VHR use. The purpose of this study was to describe PACT VHR use patterns within one VA medical center (i.e., hospital facility and five community-based outpatient clinics) pre- and post-COVID-19 onset.

## Methods and measures

This is a within-subjects study that examines changes in VHR use pre- and post-COVID-19 pandemic onset; that is, when mandatory restrictions on healthcare delivery were enacted, This study was conducted at a VA medical center in the southeastern part of the United States.

### Sampling

VHR use data from 106 individual PACTs were extracted monthly between September 2019 (baseline) and September 2020 (See Table 1). These timepoints correspond with 6 months prior to and 6 months after the onset of COVID-19-associated mandatory healthcare delivery changes. The 106 PACTs were selected based on their availability during the duration of this study, and does not reflect the current number of PACTs, or the total number of PACTs that were active at any given single timepoint. See Table 1 for a description of the PACT teamlets.

**Table 1:** Number of PACTs and number of patients within PACTs at each location

	Number of PACTs*	Panel size**	
Locations	Total *N* = 106	Mean	Range
Hospital	15	447	82–1111
Clinic 1	34	888	351–1195
Clinic 2	16	909	295–1153
Clinic 3	13	965	172–1222
Clinic 4	8	1006	861–1161
Clinic 5	8	568	231–686
Clinic 6	8	552	295–810
Clinic 7	4	872	258–1162

*Number of PACTs represents number of PACT teamlets

**Panel size represents number of Veterans cared for by a teamlet; Means and Ranges represent statistics across teamlets at a given location

### Assessed virtual resources

VA virtual care resources were made available through several online and telephonic tools. Specifically, Secure Messaging (SM) and Prescription Refills (Rx refills) were made available through a portal known as MyHealth*e*Vet (MHV). The home telehealth platform enabled video care delivery. VetLink kiosks allowed no-person contact check-ins for in-person care delivery. Finally, telephonic encounters allowed patients to connect with their PACTs.

#### VHR use variables

Use was captured in three distinct ways: 1) percent unique users of any given tool detailed below relative to the patient pool size of the PACT, referred to as *the panel size* (presented as % of panel); 2) percent encounters or messages per panel size at the PACT level (presented as % of panel); 3) rate of encounters/messages per number of unique users of any given tool (presented as number of encounters/messages:1 patient).

#### MyHealth***e***Vet Rx refills

The MHV portal allows patients to access a virtual pharmacy to reorder their prescriptions. This electronic tool is available to all patients who have a prescription written by a VA doctor that has been filled at a VA pharmacy. The MyHealth***e***Vet online features allows for tracking of patients’ past and current prescriptions, and electronic notification of shipping of prescriptions to the patients’ homes. Assessment of this construct was performed using count data of the number Rx Refills completed at the PACT level.

#### MyHealth***e***Vet secure messaging (SM)

The MHV portal allows patients to access this virtual messaging tool to communicate with their PACT. SM is available to all patients who registered, authenticated, and opted into SM. For the purpose of this study, we captured the percentage of each PACT panel at each of these steps. SM use was captured by incoming (patient initiated) and outgoing (provider initiated) secure messaging relative to panel size, as well as by number of patients in each PACT that opted in.

#### Home telehealth use

The VA Telehealth technology is a suite of technologies that allow you to meet with your care team, upload your health data for review, and develop treatment plans virtually. Telehealth technology also can be used to receive treatment and undergo exams with specialists outside of the providers in a patient’s PACT. Assessment of this construct was performed through count data of the number of telehealth visits performed at the PACT level and the number of unique patients in each PACT that utilized telehealth services.

#### VetLink kiosk use

The VetLink Kiosks are self-serve, touchscreen technological equipment that are available for use at the VA medical facility. Kiosks allow patients to electronically check-in to their appointments, review and update their personal information, and request medical records to be sent by mail. Assessment of this construct was performed through count data of the number of kiosk and clerk check-ins that were performed at the PACT level.

#### Telephone use

Telephone calls, in the form of healthcare interactions between providers and patients, represent a traditional method of VHR. Assessment of this construct was performed through both the number of PACT-level telephone visits and the number of unique patients at the PACT-level that engaged telephone visits.

### Data sources

Data were obtained from the VHA Support Service Center Capital Assets web-based project application and tracking database used for workflow and operations at the PACT level. Weekly, monthly, and annual reports provide timepoint screenshots. As approval to use SM fluctuates over time, the report generated closest to the first date of the month was used to calculate the month’s statistics. Panel size was captured from the same report. Telephone and telehealth visits, VetLink kiosk and clerk check-ins, and prescription refills were totaled for an entire month to calculate the month’s statistics. For details of data sources see data extraction blueprint in Additional file 1: Appendix 1.

### Data analysis

We checked data distribution for our outcome variables for normal distribution. Square root transformations were applied to the following variables to correct for skewness: VetLink Kiosk check-ins and Clerk check-ins. We used a piece-wise random effect model that included separate pre- and post-intervention slopes with an assumed effect size of 0.5 standard deviations from the mean [11, 12]. A sample size of 106 PACTs, given a type 1 error rate of 0.01, after Bonferroni adjustment, gave greater than 80% power for our analysis. We used the rate of use for each virtual resource as our outcome variable. Data across PACTs were standardized by their panel size (i.e., number of patients assigned to a PACT). We analyzed data from 13 monthly time points for each virtual resource, from September 2019 through September 2020, to capture VHR use 6 months prior to and 6 months after the onset of mandatory COVID-19-associated changes to healthcare delivery at the VA (i.e., March 2020).

A piece-wise random effect model was used to test change in VHR use across time as outlined in the following steps: 1) first, a time spline was computed for the timepoint that corresponds with use during March 2020. A mixed effects model tested the effect of time (13 time points) and the effect of the time spline on VHR use; we expected that the slopes before and after COVID-19 onset would be different. Models were tested and presented for the three use measures: overall rate of use across time, rate of use within PACTs, and rate of use among registered users. Models were re-run controlling for location and panel size. Tables indicated below show findings for models without these control variables given that findings did not change with the inclusion of location and panel size. The intercept represents the mean level of each of the outcomes at baseline. Estimates presented in tables are unstandardized coefficients representing the slope of change in our outcomes over time. We present two slopes: pre-COVID-19 onset slope and the difference between pre- to post- COVID-19 onset slopes, which is our main measure of change. All model parameters were estimated using restricted maximum likelihood. All results were evaluated using alpha of .05.

## Results

### PACT characteristics

During our study period (September 2019 – September 2020), we included 106 PACTs at the VA medical facility and affiliate sites; PACT panel sizes varied across teams and locations (see Table 1).

### Rate of virtual resource users pre and post COVID-19 onset

**Fig. 1:**
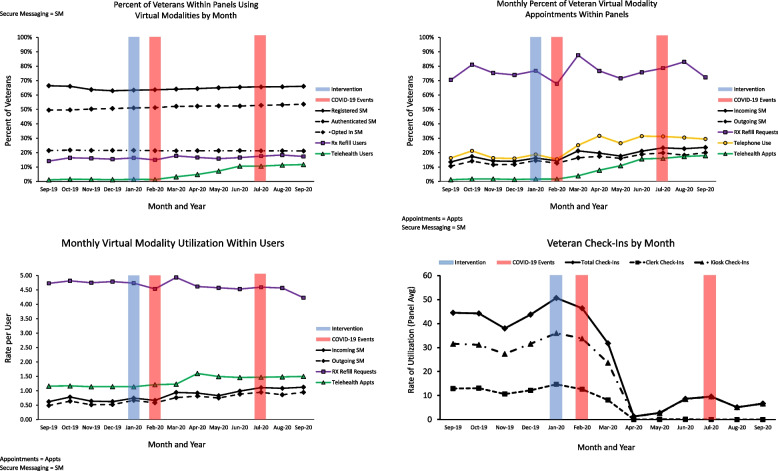
a (top left), b (top right), c (bottom left), d (bottom right): Pre and post COVID-19 onset trajectories of users and rates of utilization of virtual resources

First, we evaluated percentage of PACTs that used the identified virtual resources. We found that over the study period, on average, 66% of PACT patients were registered for SM, 49% were authenticated, and 22% opted in to use SM. Rx refills were used by 15% of PACT patients, and 10% of PACT patients utilized telehealth to communicate with their health care providers However, findings indicate that percentages of users varied across time: a comparison of the slope of rate of users before and after COVID-19 onset showed that the rate of registration increased, while the rate of authentication slightly decreased, and the rate of opting into SM remained stable. Similarly, rates of telehealth users increased after COVID-19 onset relative to the prior 6 months, while rates of Rx Refill users remained stable across time. See Table 2 and Fig. 1 a.

**Table 2:** Rate of virtual resource users before and after COVID-19 pandemic onset

Parameter	Estimate	SE	df	*t*	*p*	95% CI	
						LB	UB
Registered SM users							
Baseline Mean	.6550	.0093	102.365	70.200	.000	.6365	.6735
Pre-COVID slope	−.0045	.0003	1115.718	−15.987	.000	−.0051	−.0040
Change in slopes pre-to-post COVID	.0093	.0004	1115.866	21.944	.000	.0085	.0101
Authenticated SM users							
Baseline Mean	.4875	.0107	102.904	45.581	.000	.4663	.5087
Pre-COVID slope	.0043	.0004	1122.026	10.759	.000	.0035	.0050
Change in slopes pre-to-post COVID	−.0013	.0006	1122.146	−2.179	.030	−.0025	−.0001
Opted in SM users							
Baseline Mean	.2151	.0061	101.062	35.162	.000	.2030	.22732
Pre-COVID slope	−.0005	.0002	1120.933	−2.959	.003	−.0009	−.0002
Change in slopes pre-to-post COVID	.0002	.0003	1121.021	.760	.447	−.0003	.0007
Telehealth users							
Baseline Mean	.0970	.0072	217.005	13.429	.000	.0827	.1112
Pre-COVID slope	.0062	.0010	1082.587	5.927	.000	.0041	.0082
Change in slopes pre-to-post COVID	.0278	.0016	1083.764	17.913	.000	.0248	.0309
RxRefill users							
Baseline Mean	.1477	.0061	172.067	24.182	.000	.1356	.1598
Pre-COVID slope	.0016	.0007	1105.057	2.242	.025	.0002	.0031
Change in slopes pre-to-post COVID	.0010	.0011	1106.336	.846	.398	−.0012	.0031

Pre-COVID slope = slope of change over the course of 6 months pre-COVID related changes to care. Change in slopes pre-to-post COVID = change in slope of change over the course of 6 months post-COVID relative to 6 months slope of change pre-COVID

### Rate of VHR use pre and post COVID-19 pandemic onset within PACTs

Next, we investigated rate of actual use at the panel level. Specifically, we evaluated the rates of inbound and outbound SM, number of telephone and telehealth visits, and Rx Refill requests. We found that the rate of inbound and outbound SM and telephone and telehealth visits increased, while Rx Refill requests remained stable after COVID-19 onset relative to the prior 6 months. See Table 3 and Fig. 1 b.

**Table 3:** Rate of virtual health resource use within PACTs before and after COVID-19 pandemic onset

Parameter	Estimate	SE	df	*t*	*p*	95% CI	
						LB	UB
Inbound SM received from patients							
Baseline Mean	.1430	.0101	151.922	14.122	.000	.1230	.1630
Pre-COVID slope	.0032	.0011	1120.468	2.981	.003	.0011	.0053
Change in slopes pre-to-post COVID	.0078	.0016	1121.456	4.855	.000	.0047	.0110
Outbound SM sent by providers							
Baseline Mean	.1080	.0010	159.782	10.903	.000	.0884	.1275
Pre-COVID slope	.0052	.0011	1120.151	4.682	.000	.0030	.0074
Change in slopes pre-to-post COVID	.0037	.0017	1121.233	2.244	.025	.0005	.0070
Telephone encounters							
Baseline Mean	.3969	.0124	220.932	31.931	.000	.3724	.4213
Pre-COVID slope	.0078	.0018	1107.346	4.438	.000	.0044	.0113
Change in slopes pre-to-post COVID	.0105	.0026	1108.689	3.978	.000	.0053	.0157
Telehealth visits							
Baseline Mean	.1002	.0094	234.479	10.695	.000	.0817	.1186
Pre-COVID slope	.0084	.0014	1082.919	6.003	.000	.0057	.0111
Change in slopes pre-to-post COVID	.0356	.0021	1084.118	17.087	.000	.0315	.0397
RxRefill requests							
Baseline Mean	.7214	.0289	207.515	24.935	.000	.6643	.7784
Pre-COVID slope	.0046	.0039	1104.845	1.178	.239	−.0031	.0124
Change in slopes pre-to-post COVID	−.0018	.0059	1106.341	−.302	.763	−.0133	.0098

Pre-COVID slope = slope of change over the course of 6 months pre-COVID related changes to care. Change in slopes pre-to-post COVID = change in slope of change over the course of 6 months post-COVID relative to 6 months slope of change pre-COVID

### Rate of VHR use pre and post COVID-19 onset among users

Finally, we looked at rate of use among users only. The baseline rate of inbound SMs was .65 per 1 patient who had opted in (.65:1), and the baseline rate of outbound SMs was .49:1 (see Table 4). The baseline rate of telehealth visits among users was at 1.07:1 and Rx Refill requests among users was 4.73:1. With the exception of telehealth visits, both incoming and outgoing SM rates increased from the pre to post pandemic onset; while RxRefill requests among users showed a slight pre-post decrease for the same time period (See Table 4 and Fig. 1 c).

**Table 4:** Rate of virtual health resource use among users before and after COVID-19 pandemic onset

Parameter	Estimate	SE	df	*t*	*p*	95% CI	
						LB	UB
Inbound SM received from patients							
Baseline Mean	.6450	.0386	174.876	16.860	.000	.5739	.7261
Pre-COVID slope	.0158	.0047	1118.721	3.380	.001	.0066	.0249
Change in slopes pre-to-post COVID	.0423	.0070	1120.039	6.059	.000	.0286	.0560
Outbound SM sent by providers							
Baseline Mean	.4871	.0401	199.061	12.146	.000	.4080	.5662
Pre-COVID slope	.0260	.0053	1119.903	4.913	.000	.0156	.0364
Change in slopes pre-to-post COVID	.0200	.0079	1121.307	2.527	.012	.0045	.0355
Telehealth visits							
Baseline Mean	1.0408	.0119	811.288	87.354	.000	1.0174	1.0642
Pre-COVID slope	.0123	.0024	1107.287	5.074	.000	.0075	.0171
Change in slopes pre-to-post COVID	.0067	.0036	1107.416	1.845	.065	−.0004	.0137
RxRefill requests							
Baseline Mean	4.7333	.0679	397.655	69.723	.000	4.6000	4.867
Pre-COVID slope	.0008	.0105	1678.114	.078	.938	−.0197	.0213
Change in slopes pre-to-post COVID	−.0500	.0157	1679.618	−3.192	.001	−.0808	−.0193

Pre-COVID slope = slope of change over the course of 6 months pre-COVID related changes to care. Change in slopes pre-to-post COVID = change in slope of change over the course of 6 months post-COVID relative to 6 months slope of change pre-COVID

### Aggregate counts of VetLink kiosk and clerk check-ins pre and post COVID-19 onset

VetLink Kiosk use was collected as an indicator of in-person care use in PACTs. Clinic check-ins both at VetLink Kiosk (Estimate = −.53, Standard Error (SE) = .02, t-value = − 26.24, *p*-value < .001, 95% Confidence Interval (CI) = −.57, −.49) and by clerk (Estimate = −.60, SE = .03, t-value = − 20.52, p-value < .001, 95% CI = −.66, −.55) abruptly diminished after COVID-19 onset relative to the prior 6 months. See Fig. 1 d.

## Discussion

Since the development of VHR in the healthcare setting, implementation efforts have focused on increasing the use of VHR to increase access through remote delivery of care. Understanding the impact of the COVID-19 pandemic on VHR use can inform opportunities for using VHR to deliver care remotely. In this study we described changes in primary care delivery via VHR pre- to post- COVID-19 onset at one VA medical center. We obtained monthly VHR use rates among PACTs using secondary data extracted for the period between September 2019 and September 2020. These data provide a baseline of VHR use at the PACT level after the initial impact of the COVID-19 pandemic and can inform healthcare delivery changes within the VA systems over time. Moreover, this project produced a data extraction blueprint, that is the first of its kind to track VA VHR use leveraging secondary data sources.

While SM registration rates increased from the pre- to post-COVID-19 onset period, authentication of users decreased, and opting into SM remained stable. This suggests that while more patients had ability to access this resource, these patients were not actively electing to use SM to communicate with their PACTs. Active use of SM, in both the number of inbound and outbound messages, however, increased over time. This was true both when evaluating SM communication rates at the PACT teamlet level, and among just those users that opted into using SM. This suggests that although there was not an increase in new SM users, existing users increased their communication with their providers via this tool during the study time period. The use of technology to communicate with providers during the onset of the COVID-19 pandemic may have required some patients to learn new technological skills. Alpert et al. found in their study of SM usage during the COVID-19 pandemic that the highest percentages of patients communicating electronically via SM were between 45 and 64 years old [13]. Strategies to engage and educate older patients on the use of technology like SM may be needed to facilitate their participation in this communication tool.

A different pattern was observed for telehealth. The number of telehealth users steadily increased after the onset of the COVID-19 pandemic relative to the previous 6 months, and so did the number of telehealth encounters at the PACT teamlet level. Our findings are consistent with a scoping review on the use of telehealth during the COVID-19 pandemic which found an exponential increase in telehealth use for surveillance, triage and diagnosis, treatment including e-prescriptions, and follow-up and rehabilitation [14]. However, encounter rates among users specifically remained relatively stable. This suggests that while there was an increase in overall number of telehealth users and the overall rate of telehealth encounters at the PACT teamlet level, there was not an increase in the rate of communication among users using this tool, unlike SM users.

As the numbers of telehealth users increased, and the rates of SM use and telephonic encounters increased among users, there was an abrupt decrease in measures associated with in person care, such as VetLink Kiosk or clerk check-ins. This is consistent with the changes to healthcare delivery via telehealth and telephone encounters that were implemented on an institutional level by the VA in the face of the COVID-19 pandemic [15].

Use of virtual RxRefill remained either stable or decreased across the various assessed constructs. Both the number of users and refill requests remained stable over the study period, and refill request rates among users showed a decreasing slope post COVID-19 onset after an initial spike, suggesting a temporary increase when COVID-19 changes to care were initially announced. Our data findings are consistent with a retrospective analysis from Jan 2014 to November 2020 found that medications that require face-to-face visits had decreased during COVID-19 [16]. These findings provide a possible explanation as to why there were decreased refill rates over time.

Even prior to the COVID-19 pandemic, it appears that there has been a significant interest in healthcare delivery via virtual modalities. Findings indicate that registration for SM was high both before and after the COVID-19 pandemic, with nearly three fourths of patients being already registered for SM. This interest in using virtual modalities and opting into using virtual modalities is juxtaposed against actual use rates. SM use hovered just above 20% of PACT patients throughout the study period. However, among users, use of SM, telephone visits, and Rx Refills was stable or increased through the study period.

The virtual resource that saw the largest increase in use during the pandemic was telehealth. This finding is consistent with the suspension of all in-person encounters at the onset of the COVID-19 pandemic and the resulting changes to care that were implemented at the institutional level of the VA facility as well as globally [17]. As telehealth encounters incorporate both video and audio communication, they act as substitutes for in-person encounters. Though data indicated an increase in use among specific VHR, there is no evidence of proactive integrated use of VHR. These data present a preliminary protocol for examining the continued use, and potential emergence of proactive integrated use as providers continue to deliver remote care in a COVID-19 climate.

Study limitations should be considered when interpreting data findings. First, it should be noted this was an ongoing study when the COVID-19 pandemic occurred, as such the aim of this analysis was post-hoc in nature. Second, we evaluated changes in virtual care at one VA facility and associated community-based outpatient clinics. Locality-based characteristics, including a variety of socio-economic factors, may impact the uptake of virtual modality care in dissimilar patient populations. However, these data were not available in the extracted datasets. Third, results from some virtual modalities, VetLink Kiosk use for example, used a different PACT naming system that did not allow us to control for panel size.

Moving forward, as the VA progresses towards optimizing and proactively fully integrating virtual healthcare delivery, future research could identify implementation strategies to support use and integration, such as, address workload credit, VHR education and imbedded support for providers as they navigate VHR use challenges. Further research on barriers towards opting-into virtual modalities is needed and may include a variety of factors, such as self-efficacy with utilizing these technological tools. However, our study found that in spite of existing barriers and constraints associated with the COVID-19 pandemic, users opt into using VHR resources and demonstrated robust use. These data provide insights into virtual resource use in the context of an unprecedented pandemic. Data findings and the data extraction blueprint can be used to inform subsequent studies using a national sample or evaluating trends at rural and non-rural healthcare facilities. Future research is also required to determine if VHR use trends persist or waver post-pandemic, and if PACT members – and patients – need additional interventions to increase both uptake and proactive integrated use of virtual resources.

## Supplementary Information

**Additional file 1.** Appendix 1

## Data Availability

The datasets during and/or analyzed during the current study available from the corresponding author on reasonable request.
