# Supplementary material for: Primary care virtual resource use prior and post COVID-19 pandemic onset
Source: BMC Health Serv Res. 2022 Nov 18;22:1370. doi: 10.1186/s12913-022-08790-w (PMC9673210; doi:10.1186/s12913-022-08790-w)
Supplement: Supplementary file 1 — Additional file 1. Appendix 1 [file 12913_2022_8790_MOESM1_ESM.docx]

**APPENDIX 1**

Data Extraction Blueprint^1^

| **Construct** | **Variables** | **Measure** | **Data Source** | **Level of Granularity** | **Benefits** | **Missed Opportunities^2^** |
| --- | --- | --- | --- | --- | --- | --- |
| Secure Messaging Use | Registration | Number of patients registered | <https://reports.vssc.med.va.gov/ReportServer/Pages/ReportViewer.aspx?/SecureMessaging/SM_Statistics_Teams&rs:Command=Render> | Data is available at the teamlet level. | Data are clean and well organized making it easy to analyze with minimal effort to process the extracted data. | Data extracted provides only cumulative data, not change data between time periods. |
|  | Authentication | Number of patients authenticated | PACTCompass  VSSC_Primary Care Management Module (PCMM)  <http://vssc.med.va.gov/webrm/vssc_linksv2.aspx?PROD_ID=4363&index=1> | Data is available at the teamlet level. | Data are clean and well organized making it easy to analyze with minimal effort to process the extracted data. | Changes in teamlet makeup are not flagged over time. Data extracted provides only cumulative data, not change data between time periods. |
|  | Opted-in | Number of patients opted in |  |  |  |  |
|  | Inbound SM | Number of inbound messages |  |  |  |  |
|  | Outbound SM | Number of outbound messages |  |  |  |  |
| MHV Rx Refills Use | Prescription refill orders | Number of prescription refill orders | VSSC Transformation Initiative data cube or VCHIO data request |  |  |  |
| Telephone Use | Encounters | Number of encounters | VSSC Primary Care Management Module (PCMM)  <http://vssc.med.va.gov/webrm/vssc_linksv2.aspx?PROD_ID=4363&index=1> |  |  |  |
| Home Tele-health Use | Encounters | Number of encounters | Readily available report here: <https://reports.vssc.med.va.gov/ReportServer/Pages/ReportViewer.aspx?%2fTelehealth%2fConnectedCare&rs:Command=Render> | Facility level | Provide an overall facility summary | The only report that does not have higher granularity, such as teamlet.  Visit data was not distinguished from encounter data, although VA use of the terms differs. |
|  | Visits^3^ | Number of visits |  |  |  |  |
|  | Unique Patients | Number of unique patients |  |  |  |  |
|  |  |  | CDW Telehealth Visits Report, including secondary codes for Home Telehealth, Clinical Video, Store & Forward | Data can be extracted at the patient, provider, teamlet levels | High granularity of data | This process involved regulatory steps, such as QI determination or IRB approval for research, DART request approval for data provision |
| VetLink kiosk Use | Check-in | Number of patients checked-in | Here is the link to request the access to VETLINK:  <https://r03tamappvps01.r03.med.va.gov/vCas/login?service=https%3A%2F%2Fr03tamappvps01.r03.med.va.gov%2Fkiosk-workflows%2Fadmin%2Fj_spring_cas_security_check> | Kiosk Group (multiple clinics) or Clinic (multiple teamlets) | Easy to use by importing directly from the kiosk portal. Tables are formatted and require some processing to prepare for analysis. | Patient or teamlet level data is available for pay at [vetlink-orders@vecna.com](mailto:vetlink-orders@vecna.com). Changes to clinics are not flagged. Changes to Kiosk groups are only visible if clinic level data are extracted. Demographic update and Assistance Required data available at the location level not pact level for Riverview, Zephyrhills, NPR, Brooksville – therefore numbers represent use for PACT + other OPC at these locations. |
|  | Demographic update^3^ | Number of patients who updated demographic data |  |  |  |  |
|  | Assistance Required^3^ | Number of patients who required assistance at kiosk |  |  |  |  |

Notes:

^1^ All data has been pulled for the period of September 2019 - September 2020

^2^A missed opportunity across all the variables was the ability to captured data directly from the Data Acquisition & Analytic Service. As a team that regularly captures this data for the Tampa VA for workflow purposes, they have capabilities for capturing more granular data, and precise knowledge about the specifics of data management, which are invaluable skills for data extraction and cleaning. This resource is available for clinician and administrative purposes, but currently is unavailable for research purposes.

^3^Data was not used for our analysis purposes due to lack of availability of granular data.
